# Magnet therapy for the relief of pain and inflammation in rheumatoid arthritis (CAMBRA): A randomised placebo-controlled crossover trial

**DOI:** 10.1186/1745-6215-9-53

**Published:** 2008-09-12

**Authors:** Stewart J Richmond

**Affiliations:** 1 Department of Health Sciences, The University of York, England, UK

## Abstract

**Background:**

Rheumatoid arthritis is a common inflammatory autoimmune disease. Although disease activity may be managed effectively with prescription drugs, unproven treatments such as magnet therapy are sometimes used as an adjunct for pain control. Therapeutic devices incorporating permanent magnets are widely available and easy to use. Magnets may also be perceived as a more natural and less harmful alternative to analgesic compounds. Of interest to health service researchers is the possibility that magnet therapy might help to reduce the economic burden of managing chronic musculoskeletal disorders. Magnets are extremely cheap to manufacture and prolonged treatment involves a single cost. Despite this, good quality scientific evidence concerning the safety, effectiveness and cost-effectiveness of magnet therapy is scarce. The primary aim of the CAMBRA trial is to investigate the effectiveness of magnet therapy for relieving pain and inflammation in rheumatoid arthritis.

**Methods/Design:**

The CAMBRA trial employs a randomised double-blind placebo-controlled crossover design. Participant will each wear four devices: a commercially available magnetic wrist strap; an attenuated wrist strap; a demagnetised wrist strap; and a copper bracelet. Device will be allocated in a randomised sequence and each worn for five weeks. The four treatment phases will be separated by wash out periods lasting one week. Both participants and researchers will be blind, as far as feasible, to the allocation of experimental and control devices. In total 69 participants will be recruited from general practices within the UK. Eligible patients will have a verified diagnosis of rheumatoid arthritis that is being managed using drugs, and will be experiencing chronic pain. Outcomes measured will include pain, inflammation, disease activity, physical function, medication use, affect, and health related costs. Data will be collected using questionnaires, diaries, manual pill counts and blood tests.

**Discussion:**

Magnetism is an inherent property of experimental devices which is hard to conceal. The use of multiple control devices, including a copper bracelet, represents a concerted attempt to overcome methodological limitations associated with trials in this field. The trial began in July 2007. At the time of submission (August 2008) recruitment has finished, with 70 trial participants, and data collection is almost complete.

**Trial Registration:**

Current Controlled Trials ISRCTN51459023

## Background

### Need for a trial

Rheumatoid arthritis (RA) is a chronic inflammatory autoimmune disease which typically begins to develop between 30 to 50 years of age. It is estimated that 387,000 people in the UK have RA, with three times more women affected than men. RA involves periodic inflammation of the synovium in joints of the hand, wrist, foot, knee or shoulder. This causes swelling of the joint capsule and irritation of nerve endings, producing pain and resulting in damage to both bone and cartilage. In turn this may lead to both disability and mortality [1,2]. Favoured diagnostic criteria are those of the American College of Rheumatology [3].

The burden which RA imposes upon both the UK National Health Service (NHS) and the economy in general is considerable. It is reported that over just a one year period (1999 to 2000) inflammatory arthritis accounted for 1.9 million GP consultations, 45,887 hospital admissions and a loss of £833 million in productivity [1]. For the individual the consequences of RA may be devastating.

Treatment of RA is currently dominated by the use of medication. Non steroidal anti-inflammatory drugs (NSAIDs), including second generation Cox-2 inhibitors, together with disease modifying anti-rheumatic drugs (DMARDs) are the most widely used pharmacological agents for managing RA [4]. However recently developed biological agents, including TNF-α antagonists, have also been found to prevent damage and are becoming more widely prescribed [5]. Whilst such drugs may alter disease progression, people with arthritis rarely find that they are entirely adequate for the purpose of pain control. Therefore analgesics are also typically prescribed as an adjunct [6].

The fact that all commonly prescribed drugs for rheumatoid arthritis are associated with unwanted side effects is worrying for both practitioners and patients. For example, DMARDs are associated with high levels of toxicity and therefore cannot be tolerated for long periods [5]. Moreover, NSAIDs have been shown to cause gastrointestinal ulcers, which may result in perforation, bleeding and death [4,7,8].

Over recent years health professionals have witnessed a patient led revolution in the use of complementary and alternative medicine (CAM). Whilst reliable estimates for CAM use are hard to obtain, results from a fairly recent population based survey indicated that around 28% of the UK adult population use some form of CAM in just one year [9]. However, it has also been shown that people who experience chronic pain are twice as likely to try CAM [10]. One might therefore anticipate that patients with RA would be included amongst this group. Indeed, this appears consistent with suggestions that almost 60% of people with arthritis use complementary therapy [11]. In part, such popularity may also be attributed to the perception, albeit sometimes misguided, that complementary therapies lack side effects and therefore represent a safe and 'natural' alternative to drugs [4,12]. Alternatively this may simply relate to a rise in holistic attitudes [10,13].

Whatever the case, such treatments have typically been viewed with scepticism by the medical community. Yet assumptions which directly equate lack of previous scientific evidence to lack of therapeutic efficacy should be challenged. Rather attention should be drawn to the fact that until recently CAM accounted for less than 1% of total research budgets [14]. This being a figure which is grossly disproportionate to the measurable contribution which CAM makes towards first-contact primary care [9]. With a growing emphasis on patient choice [15], CAM is now becoming recognised as a largely untapped resource which offers the potential for therapeutic innovation within the NHS. This view is supported by both the House of Lords and the UK government, which have recommended a major increase in funding for CAM research [13,16].

Perhaps one of the most intriguing yet under researched forms of CAM is that of magnet therapy. Quite simply it involves the application of magnetic materials on or very close to the skin over prolonged periods of time. This encompasses a wide range of interventions involving different types of devices, different strength magnetic fields and different modes of administration [17]. For example, devices may be unipolar, in which case only one magnetic pole faces towards the skin, or bipolar when two or more magnets are used which face in opposite directions [18]. However magnet therapy should not be confused with pulsed magnetic field therapy, which involves brief exposure to much stronger electromagnetic fields.

Fascination with the potential health benefits of magnetic forces dates back to antiquity, although in recent times this topic has received a resurgence of public interest [19]. Magnet therapy now appears to be one of the most widely used forms of CAM for the management of chronic pain associated with musculoskeletal disorders such as RA, with many patients demonstrating willingness to privately purchase permanent magnetic devices which are marketed for health purposes. Indeed, it has been estimated that the worldwide sales of such devices account for somewhere between one and four billion US dollars each year [20,21].

Whilst such devices are widely claimed to help alleviate pain for a range of disorders, there appears to be no clearly identified mechanism of action, although theoretically this might involve a reduction in inflammation [18]. Scientific evidence relating to the effectiveness of magnet therapy is however reported to be equivocal [22]. This has lead to calls for larger and more rigorous randomised controlled trials (RCTs) to determine the actual therapeutic effectiveness, safety and cost-effectiveness of magnet therapy for specific pathological conditions [17,23].

### The placebo problem

Perhaps the greatest methodological challenge faced by trials of magnet therapy is that of providing adequate control devices against which any 'true' effects from magnet therapy may be assessed. RCTs which incorporate a placebo control group are generally perceived as methodologically superior to those which do not. This is especially the case for trials involving new treatments and subjective outcome measures [24,25]. The key methodological advantage of placebo controlled trials over open trial designs relates to the ability to blind participants, clinicians and researchers to the allocation of experimental and control treatments [26]. This helps to avoid systematic bias resulting from differences in the perceived or desired effectiveness of treatments which may alter the outcomes being assessed [27-29].

For trials of magnet therapy successful blinding is hampered by the fact that magnetism is a necessary quality of experimental devices which can in most cases be detected with relative ease. Trials which report superior patient outcomes for a magnetic device as opposed to a non-magnetic control device are therefore likely to be criticised on the basis that results may be attributable to non-specific (placebo) effects [30,31]. This highlights the need to evaluate success of blinding [25].

The problem of blinding is not necessarily without solution however. One approach is to use an attenuated control device, which retains a weak magnetic force and therefore helps to prevent participants and researchers from correctly identifying the device as a placebo or sham [32,33]. One limitation of this approach however is that rather than being completely inert the control device may have some therapeutic action. As such, this may produce a more conservative estimate of treatment effect between groups.

An alternative approach, yet to be reported, is to use a non-magnetic comparator which is marketed commercially as having similar therapeutic benefits, but which in truth is ineffective. Such a device would need demonstrate equivalent face validity, i.e. be perceived as equally credible in terms of anticipated therapeutic effects. It should also be used in a similar fashion to the experimental magnetic device. For trials which seek to evaluate magnetic wrist straps, copper bracelets might serve as a suitable placebo.

### Copper bracelets

Copper bracelets are often worn by people who suffer with arthritis in the belief that they may help to prevent inflammation and pain. Whilst research findings point towards serum copper imbalances in patients with arthritis [34-36], it is likely that this reflects a natural metabolic response, rather than a cause of inflammation [37]. A proposed link between dermal copper absorption and pain relief in arthritis may therefore be rather tenuous. However, given the popular use of copper bracelets it is surprising that no rigorous scientific research appears to have been conducted on the effectiveness of such devices.

In the most widely cited study on this topic, Walker and Keats randomised 240 arthritis sufferers into three groups [[38], see also [39]]. Group 1 wore a copper bracelet for one month and then an aluminium bracelet for a further month. Group 2 wore identical devices but in reverse order and Group 3 wore no device. From this Walker and Keats reported that significantly more participants rated the copper bracelet as superior than the aluminium bracelet and that copper bracelets actually lost weight by an average of 13 mg/month. This appears to support the theory that copper may be leached into the skin and that this may have had a positive therapeutic effect on arthritis symptoms. However, Walker and Keats failed to demonstrate any positive correlation between the weight loss of individual bracelets and perceived efficacy. Major criticisms of this study centre on selection bias and measurement error. For example, 163 or 68% of all participants were excluded from the analysis. Some of whom were removed due to poor compliance, whilst others were excluded on the grounds that their bracelets were observed to have gained weight. It is not surprising therefore that the remaining bracelets included in the analysis were reported to have lost weight. Blinding is also likely to have been compromised since the control bracelet would have appeared different in weight, colour and corrosion. As such, these findings have yet to be replicated elsewhere.

More compelling evidence to refute a possible link between dermal copper absorption and pain relief in arthritis comes from Shackel and colleagues, who reported findings from a seemingly well conducted double-blind placebo-controlled trial [40]. In this study 116 participants were randomly allocated to receive either copper-salicylate gel or a placebo gel, which they applied daily over a one month treatment period. Whilst one would expect copper to be absorbed more readily from a topical preparation than a copper bracelet, findings from this trial failed to show any difference between the copper-salicylate gel and the placebo gel in terms of pain relief, ratings of treatment efficacy or medication use. Yet the extent to which these findings may be generalised to patients with RA also is open to question.

### Systematic reviews

A review of scientific databases (Cochrane Library, Medline, AMED and CINAHL) up to July 2007 confirmed an overall paucity of rigorous scientific research into the therapeutic effectiveness of magnet therapy for RA, or any other specific disorder. Whilst a number of critical reviews have been published concerning magnet therapy [e.g. [17,41]], these have attempted to answer the same basic question, i.e. whether or not magnet therapy is effective for pain control. Such reviews have therefore tried to combine data relating to a wide spectrum of different pain related disorders and have also incorporated studies in which pain has been experimentally induced. Moreover, major inconsistencies between studies in terms of intervention (e.g. according to the magnetic strength of devices used and also exposure periods involved) has further served to obscure any clear findings. As yet no obvious attempt has been made to undertake a systematic review specifically concerned with the effects of magnet therapy for people with arthritis.

### Previous trials of magnet therapy for rheumatoid arthritis

To the authors' knowledge only one previous RCT has focused specifically on the effects of magnet therapy for patients with RA. Segal and colleagues randomly allocated 64 patients with RA of knee to wear either a bipolar magnetic device (MagnaBloc^®^) or a seemingly identical unipolar device [32]. The experimental device consisted of four magnets disks (each 1900 Oersteds) whereas the control device included just one magnetic disk (720 Oersteds) and three non-magnetic disks. Devices were attached to the knee for a period of one week in both the treatment (n = 38) and control (n = 26) groups. Both participants and researchers were reportedly blind to treatment allocation. Although participants allocated to the MagnaBloc^® ^group demonstrated a statistically greater reduction in pain throughout the intervention phase than those in the control group, the study failed to demonstrate a statistically significant difference in pain between the two groups at the end of the 1 week period (p = 0.23). In terms of surrogate measures of inflammation (C-reactive protein and erythrocyte sedimentation rate) no difference was observed between treatment and control groups. In the absence of any evaluation of success in blinding, it is interesting to note that participants global assessment of disease activity improved to a significantly greater extent in the treatment group than in the control group (p < 0.01).

Whilst results presented by Segal and colleagues may be challenged on the basis of poor trial design and lack of statistical power, this study nevertheless highlights a number of other methodological weaknesses which characterise other published trials of magnet therapy. Importantly, medication use was not measured although participants were likely to alter their use of analgesics as a response to changes in pain intensity. Any true benefit derived from magnet therapy may therefore have been masked by a reduction in medication use. In order to provide a pragmatic evaluation of effectiveness, trials of magnet therapy should offer participants the opportunity to use devices in a manner which reflects real world behaviour. Since magnet therapy is generally understood to involve prolonged periods of exposure to magnetic fields, any short term experimental intervention may prove insufficient in order to demonstrate a cumulative therapeutic effect. In the case of the trial described by Segal and colleagues, an exposure period of just one week may well have been inadequate. Information on compliance was also omitted, providing little assurance that trial participants actually wore the devices they were given. Even if data are to be analysed by intention to treat rather than by active treatment, information concerning compliance will still be of value.

### Pilot trial (MACROPOD)

The proposed study builds upon methodology employed in an earlier RCT, referred to as the MACROPOD trial, which was designed and led by the present author.

MACROPOD (ISRCTN 18518978) was a randomised double-blind placebo-controlled crossover trial of magnet therapy for pain relief in osteoarthritis. In total 45 participants were randomly allocated to one of four treatment sequences consisting of four phases, with each phase lasting one month. Participants each wore a full strength bipolar magnetic bracelet (mean strength 2009 Oersteds, SD 177), an attenuated bracelet (453 Oersteds, SD 120), a non-magnetic bracelet and a copper bracelet. Outcomes measures included the WOMAC 3.1 Osteoarthritis Index (which covers pain, physical function and stiffness), for which the pain subscale was selected as the primary outcome measure. The trial also incorporated the McGill Pain Questionnaire and the EQ-5D. Medication use was calculated by triangulation of diary records, prescription records and by manually counting remaining tablets and capsules before and after each phase. Economic data concerning the use of health care resources were collected in order to facilitate future cost-effectiveness analyses.

Whilst originally conceived as a pilot RCT, MACROPOD was nevertheless developed in order to achieve sufficient statistical power to demonstrate a meaningful clinical difference in pain outcomes between the commercially available magnetic device and the three 'placebo' devices.

Findings from the MACROPOD trial have been summarised elsewhere [42] and are currently being prepared for publication in greater detail. In terms of methodology, the MACROPOD trial served to demonstrate feasibility for a larger and somewhat more comprehensive RCT. In particular, the use of multiple control devices appeared to represent a useful and valid approach in helping to address the problem of adequate placebo control. It is nevertheless worthwhile noting that MACROPOD concerned osteoarthritis rather than rheumatoid arthritis, and that no biological outcome measures were employed.

### Ongoing trials

A search of The Cochrane Central Register of Controlled Trials (CENTRAL) and the *meta*Register of Controlled Trials (*m*RCT) conducted in July 2007 indicated that no other RCTs of magnet therapy for arthritis were in progress at this time.

### Objectives

The proposed trial will investigate the therapeutic effectiveness of magnet therapy when used as an adjunct to practitioner led management of pain and inflammation in rheumatoid arthritis. Possible effects on other major health outcomes will also be considered. This will involve a comparison of treatment effects between a commercially available magnetic device and three control devices, which are intended to act as placebos. This addresses a need for rigorous scientific evidence on the subject.

#### General objective

To investigate the therapeutic effects of wearing a widely available permanent static magnetic device for patients with RA.

#### Specific objectives

1. To determine the actual therapeutic effectiveness of a magnetic wrist strap in terms of the following outcomes:

• pain

• inflammation

• functional status

• general health

• quality of life

• use of medication

Note: Specific outcomes assessed will be used to provide a composite measure of disease activity.

2. To identify specific factors that may predict or mediate therapeutic effects of magnet therapy.

3. To identify any specific factors which mediate observed placebo effects.

4. To evaluate the therapeutic effectiveness of copper bracelets, and their potential role as placebos in magnet therapy research.

5. To report any adverse events or reactions relating to the use of devices employed.

6. To gather economic data that could be used to evaluate the cost-effectiveness of magnet therapy.

## Methods

### Design

The trial will employ a randomised crossover design. Therefore each participant will act as his or her own control. The principle advantage of a crossover design is that error variance is reduced, thereby minimising the sample size required. Crossover designs are also extremely well suited to exploratory research of new treatments [43].

### Population

Patients will be suffering with rheumatoid arthritis and also receiving active treatment within a primary care setting.

#### Inclusion criteria

1. 18 years of age or over.

2. Diagnosis of rheumatoid arthritis within medical records.

3. Currently prescribed analgesic medication, NSAIDs or DMARDs.

4. Chronic pain: either persistent or intermittent over a minimum period of three months prior to recruitment [44].

5. Current pain: greater than 30/100 mm on a pain visual analogue scale (VAS) within the last 24 hours despite medication.

#### Exclusion criteria

1. Pregnancy.

2. Pacemaker or similar device.

3. Not responsible for administering his/her own medication.

4. Dementia or memory impairment, either documented in medical records or if suspected indicated by a score of 6 or below on the Abbreviated Mental Test [45].

5. Diagnosis of malignant disease within medical records.

6. Forthcoming orthopaedic surgery within the next 25 weeks.

7. Known allergy to copper.

8. Regular use of a magnetic or copper device at recruitment.

Within the proposed trial it would neither be desirable, practical nor ethical to limit recruitment to patients who are either not prescribed, refuse or stop drug treatments in order to participate in the study. In particular, the trial attempts to evaluate the effectiveness (rather than efficacy) of magnet therapy within a real world context. Most people with verified and active RA will be taking medication. Recruitment of sufficient patient numbers would therefore prove extremely difficult if those taking medication were excluded. This would also involving a highly selective population, severely limiting the generalisability of results. Moreover, even those who are not taking medication at the start of such a study may well alter their status during the trial. It is also likely to be viewed as unethical to ask patients to stop taking their medication in order to take part in the trial. This is because the devices involved have largely unproven therapeutic effects, are not currently thought to alter disease progression, and because multiple placebo devices will be used.

### Interventions

Given the inherent difficulties in developing a 'perfect' placebo for magnet therapy, this study will build upon methodology employed within the MACROPOD trial by incorporating one experimental and three distinct control devices. The use of multiple control devices represents a comprehensive and concerted attempt to overcome different limitations associated with each individual device in terms of blinding and potential therapeutic action. All participants will therefore undergo four treatment phases:

#### Full strength MagnaMax^® ^(experimental device)

The experimental device selected for use in this trial is the MagnaMax^® ^magnetic wrist strap. This is a widely available magnetic device which is marketed for the purpose of relieving pain in conditions such as rheumatoid arthritis. It is representative of other commonly purchased devices in terms of its application and magnetic strength. This device consists of two concentric neodymium magnets which fit together into a bracelet that is worn around the wrist. The central magnet can be rotated 360 degrees within the outer magnet. Similarly the outer magnet can be reversed so that there are four possible configurations for direct contact with the skin (+++, +-+, ---, -+-). Inserts will be supplied to participants in an opposing configuration. Both magnets are plastic coated, and the overall size of the insert is 23 mm in diameter. A calibrated Hall effect probe will be used to check the maximum magnetic surface field strength of the inserts.

#### Attenuated MagnaMax^® ^(placebo)

This will be identical to the experimental device with the exception that the magnetic field strength of inserts will be reduced to between 250 to 350 Oersteds. The use of a lower limit of 250 Oersteds should ensure that the device retains sufficient magnetism to firmly adhere to a vertical metal surface (e.g. a refrigerator). Participants are unlikely to identify this as a placebo, although it is possible that the weak magnetic field produced may have some therapeutic effect.

#### Demagnetised MagnaMax^® ^(dummy)

This will appear identical to the experimental device although the inserts will be demagnetised, and should therefore be entirely inert. However participants may easily identify the device as non-magnetic and therefore view it as a placebo.

#### Copper bracelet (placebo)

Participants will also test a commercially available copper bracelet. As copper bracelets are widely used for pain relief by the general public it is thought that participants may expect this to have some therapeutic benefit. Whether or not this device actually represents a valid placebo is a question which will be addressed as part of the trial.

Participants will be required to wear each of these devices for a minimum of 12 hours per day, although continuous use will be recommended. Participants will begin the first treatment phase immediately after recruitment. Each treatment phase will last five weeks and be separated by a one week 'wash out' period. Total duration for participation will therefore be 23 weeks.

Non-compliance in terms of failure to wear devices for the recommended amount of time is unlikely to represent a major problem. Compliance will be measured by asking participants how long they wore each device for and systematic bias should be avoided due to the random allocation of participants to different intervention sequences. Whilst the present study employs placebo-control, the emphasis is nevertheless that of a pragmatic evaluation of the effectiveness, rather than efficacy, of magnet therapy within a real world setting [46]. Non-compliance therefore represents a valid outcome in its own right.

### Randomisation

**Figure 1:**
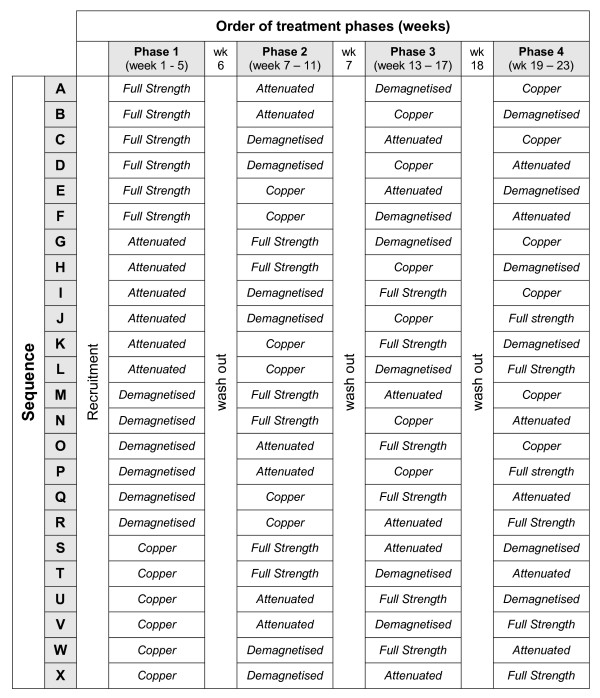
**Sequences for device allocation**. All 24 possible unique treatment sequences for the distribution of the four study devices. Each participant will be randomly allocated to one of these sequences, with the actual randomisation key being withheld from the principal investigator until completion of all data collection.

Participants will be randomly allocated to 1 of 24 possible treatment sequences. Treatment sequences will consist of four phases, with each phase corresponding to a particular device (as shown in Figure 1). This method is preferred over the use of a Latin square design, as this will help to minimise possible analytical bias associated with attrition.

Randomisation will be performed remotely and independently by staff at the York Trials Unit using a relevant computer programme to avoid any bias in sequence allocation. Randomisation will be performed using block sizes of 24, which should help to provide relatively even balance in terms of the number of people allocated to each treatment sequence. The randomisation code will be concealed from the principal investigator until all data collection has been completed. The randomisation method therefore provides little scope for subversion of the allocation process. Since each participant will test all four devices there is also no meaningful purpose for subversion. Once the full randomisation sequence for all potential participants has been determined, then devices will be packed by a member of the York Trials Unit and sealed in identical padded boxes, distinguishable only by a trial ID number and order in which they are to be distributed.

### Methods for protecting against bias

Several methods will be employed to counter additional threats from bias in terms of both the internal and external validity.

#### Blinding

Patients, GPs, and other health care staff will be offered limited information about the trial. The trial will be presented as a study to evaluate the effectiveness of magnetic and copper bracelets on arthritis symptoms. All communication will simply state that one or more of the devices being tested might be intended to act as a placebo. At no point will they be provided with specific information concerning devices or their expected effects. Moreover participants will not be given the opportunity to directly compare devices.

The principal investigator will remain blind to the order in which devices are allocated until after the completion of data collection. Devices will be independently packaged and sealed within plain white padded boxes by the York Trials Unit. Boxes will simply bear the trial ID number and order of distribution. Devices will then be distributed by the principal investigator at recruitment and at each follow-up. Each box will contain unique laser labels bearing the trial ID number and the mark of an official stamp. At the end of each treatment phase participants will be instructed to remove the device before being visited by the principal investigator and to seal the device within the padded box, using labels provided. Devices will be collected by the researcher at the end of each visit (i.e. after data collection) and returned to the trials unit. The integrity of each seal will be independently inspected and recorded. Participants will also be instructed in writing to avoid describing the device(s) they have tested in any communication with the researcher. This procedure will also help to ensure that all devices are returned, thereby preventing possible contamination of treatment effects.

#### Stratified random sampling

Roughly equal numbers of patients with RA should be approached by each participating practice. Should the number of potentially eligible patients identified within each practice exceed the target number (i.e. 52 per practice) by more than 50% (i.e. 78) then patients will be selected at random to receive information about the trial. In such case, patient recruitment will involve stratified sampling according to general practice. This should help to achieve a fairly representative sociodemographic cross section of the clinical population, which will add to the generalisability of findings.

#### Wash out-periods

Whilst randomisation of participants to different allocation sequences, together with the collection of outcome data at the end of each treatment phase, should help to prevent bias associated with potential carry over effects, wash-out periods between treatments will nevertheless be employed. As such, each treatment phase will be separated by a period of one week during which no device will be worn. Wash out periods of one week were selected as this seemed a reasonable duration by which one might expect the effects of wearing a magnetic or copper bracelet to wear off. This is also consistent with a previous randomised crossover trial concerning the effects of static magnets on low back pain, which used one week wash out periods [47]. Together this should provide additional confidence relating to the internal validity of findings and will aid in the analysis of data [43].

### Methods of data collection

Participants will be visited at home by the principal investigator on five occasions. Baseline data will be collected at recruitment. Participants will then be followed up on four further occasions, separated by intervals of six weeks. These will occur at the end of each treatment phase.

Questionnaires will be sent to participants two to three days before each planned follow up and then collected by the principal investigator. Each trial participant will be given a medication diary for each treatment phase which they will be asked to complete on a daily basis. With the participants help and prior consent (obtained at recruitment) a 'pill count' will be conducted. This will involve counting the remaining number of tablets or capsules that the participant has in their possession for each analgesic, NSAID or DMARD received on repeat prescription. Issue dates will also be recorded. At a later stage prescription records will be obtained from each participant's general practice. Information from medication diaries, pill counts and prescription histories will then be triangulated to estimate medication use during each treatment phase. This approach has been used successfully by both the RESPECT trial and the MACROPOD trial [45,42].

Participants will be required to provide blood samples on four separate occasions. Blood samples will be collected by a nurse, phlebotomist or health care assistant located within the practice, and should be taken within a two day period either side of each planned follow-up. Administrative staff within participating general practices will be responsible for organising these appointments and sending samples for analysis. Samples will be sent via normal NHS channels to local haematology laboratories for testing. Relevant trial labels will be provided for sample bottles and request cards. Results will be entered into the patient's medical records and a copy sent to the principal investigator. Once recruited, all participants will be followed up regardless of any failure to provide blood samples.

Practice staff will be encouraged to report any adverse events or reactions which could be associated with wearing study devices. Similarly, participants will be asked to report such events directly to the trial and inform their GP.

Following all other data collection activities, each practice will be visited by the principal investigator during which prescription histories and any missing data from blood tests will be obtained. Participants will be required to provide consent for access to medical records and the principal investigator will obtain honorary contracts for each of the primary care trusts (PCTs) concerned.

### Outcome measures

The recommended approach for assessment of outcomes in clinical trials of treatments for RA is to examine change according to a 'core set' of disease activity measures defined by the American College of Rheumatology (ACR) [3,48-50]. This consists of seven measures:

1. Tender joint count

2. Swollen joint count

3. Physician's global assessment of disease activity

4. Patient's global assessment of disease activity

5. Patient's assessment of physical function

6. Patient's assessment of pain

7. Acute phase reactant value

Three of these are self report measures (i.e. pain, physical function and patient's global assessment of disease activity). Whilst it has shown that a pooled index of these three measures may discriminate equally well to the 'core set' in terms evaluating the efficacy of treatments in placebo controlled trials [51], data collection will nevertheless be guided by the full core set of measures.

Three measures rely on clinical examination (i.e. swollen joint count, tender joint count and professional assessment of disease activity). However for the CAMBRA trial, repeated clinical assessments by a suitably qualified clinician (i.e. rheumatologist) are not feasible. Instead, participants will be asked to conduct self-assessed counts of tender and swollen joints using pictorial mannequins which have been validated in previous research concerning rheumatoid arthritis [52]. These images will be reproduced by permission of the relevant authors. Similarly, a professional assessment of overall disease activity must be discounted. Participants will therefore be relied upon to provide their own global assessment of disease activity using a visual analogue scale adapted from the standardised ACR patient assessment form. This will ask participants to consider all the ways that their arthritis affects them at the present time and to indicate how well they think they are doing on a 100 mm VAS accompanied by verbal anchors, with *"very well" *at one end and *"very poor" *at the other.

Changes in physical function will be assessed using the Health Assessment Questionnaire – Disability Index, which is a widely used and validated outcome measure.

Perhaps more importantly, the primary focus of this trial is on the effects of magnet therapy on pain and inflammation in RA rather than overall disease activity. As such, the primary outcome measure should be pain specific. For the present trial a 100 mm pain visual analogue scale was selected, as consistent with the recommended method of pain measurement according to ACR criteria. This displays verbal anchors indicating "no pain" at one end to "worst pain ever" at the other. This will be worded as follows: *"How much pain have you had because of your rheumatoid arthritis over the past week. Place a mark on the line below to show how severe your pain has been (0 means "no pain" and 100 means "worst pain ever")"*. Visual analogue scales have been widely used for quantifying pain for a range of conditions including rheumatoid arthritis and have been shown to be adequate for this purpose in terms of reliability, validity and sensitivity [53-55]. The McGill Pain Questionnaire will be used as a second pain outcome measure, as this covers affective components and may also be used to validate findings from the VAS. This will also aid comparison of findings with studies reported elsewhere.

Possible variation in participants' use of medication may have a confounding effect on pain outcomes. This will have the tendency of reducing any otherwise observable difference between groups resulting from a true treatment effect. Medication use during each treatment phase will therefore be quantified. This will enable pain outcomes to be adjusted if necessary on the basis of regression analysis. Whilst the main emphasis will be placed on measuring changes in the use of prescribed analgesic drugs (e.g. cocodamol and paracetamol), it is important to note that use of other drug types (i.e. NSAIDs and DMARDs) will have an impact on pain and therefore will be accounted for also.

The final measure recommended by the ARC for evaluating changes in disease activity concerns the analysis of acute phase reactants. Mechanisms involved in the alleviation of pain in RA include reduction in inflammation of the synovium. Widely used surrogate measures of inflammation include blood tests, typically C-reactive protein (CRP) and erythrocyte sedimentation rate (ESR). These are referred to by the ACR as acute phase reactants. Together these represent favoured outcome measures for monitoring disease activity [49]. Results presented by Segal and colleagues appear to show that ESR may show wider variability [32], and therefore provide greater discriminative power in terms of detecting treatment effects. However plasma viscosity (PV) has now replaced ESR within the local region in which the CAMBRA trial will be conducted. PV may be considered as largely equivalent to ESR, yet PV provides the advantage that blood samples do not need to be processed as quickly as with ESR. Given limitations associated with the sensitivity and specificity of individual tests, both PV and CRP tests will be conducted as proxies for inflammation within the proposed trial.

Besides disease activity, additional therapeutic outcomes are also of interest. In particular, the use of magnetic and copper bracelets may have important consequences on a patient's ability to cope and their emotional wellbeing. The trial will therefore incorporate both the 5-item helplessness subscale of the Arthritis Helplessness Index [56], using adapted 6-point measurement scales, and the Hospital Anxiety and Depression Index as established measures of coping and affect.

The possibility that magnet therapy may alleviate pain in chronic musculoskeletal conditions via endogenous opiod channels has also been considered. However the decision has been made not use blood samples for the purpose of exploring changes in endogenous opiods, such as beta-endorphin. This is due a number of factors, including the fact that endorphin tests are not routinely used within the NHS and therefore costs are prohibitive. Moreover, the requirement for additional blood samples involving non-routine tests is likely to be viewed as unacceptable by research participants.

The EQ-5D (EuroQol) will be used as a generic health outcome measure [57]. For the proposed trial this represent a valid outcome measures in its own right and will aid comparison of findings with other studies, such as MACROPOD. Participants will also be asked to report on their use of health care resources and personal expenditure relating to health care. This together with data collected using the EQ-5D will facilitate any cost-effectiveness analysis of magnet therapy arising from this trail.

Participants will be asked to report on compliance in terms of time spent wearing each device, indicating the number of days and hours that each device was worn. This may relate to treatment effectiveness and assurance will be required that participants actually wore the devices they were given. However poor compliance will not be used as a basis for exclusion from analyses.

Anticipated therapeutic effects from wearing magnetic and copper bracelets will be measured at baseline and compared in order to determine whether devices may be considered equivalent in terms of perceived benefits. Participants will be asked for both copper and magnetic bracelets in turn *"How likely do you think it is that wearing a *[device] *will help to relieve symptoms of your arthritis"*. Responses will be measured using 5-point bipolar Likert type scales, incorporating the following verbal anchors: *"Very unlikely"*;*"Fairly unlikely"*;*Can't decide"*;*"Fairly likely"*;*and "Very likely"*.

Success of blinding will be assessed by asking participants to judge whether or not each device worn was a placebo. In order to ensure that participants understand the question, they will first be provided with two applied definitions of what constitutes a placebo. Definition 1: *"In this study a placebo is an inactive bracelet that has no therapeutic effect, other than that caused by fooling the mind into thinking it works"*. Definition 2: *"A placebo can be thought of as a 'fake' or 'dummy' bracelet, which is used for comparison in order to measure the real treatment effect of a genuine (i.e. active) therapeutic device"*. Participants will then be asked *"Do you believe that the device you were asked to wear over the past 5 weeks was a placebo?"*. Responses will be measured using a 5-point bipolar Likert type scale, displaying the following verbal anchors: *"Definitely a placebo (i.e. inactive)"*; *"Probably a placebo (i.e. inactive)"*; *"Can't decide"*; *"Probably not a placebo (i.e. active)"*; and *"Definitely not a placebo (i.e. active)"*. One limitation of this approach is that participants may differentiate between devices based purely on perceived effects, rather than any consideration of the device itself. A device which has a clear and genuine therapeutic effect is therefore less likely to be judged as a placebo than a device which has no apparent effect, regardless of the adequacy of blinding procedures. Participants will therefore also be questioned after completing the trial, as to whether they noticed any differences between devices (e.g. in terms of magnetism).

Adverse events and reactions will be monitored throughout the trial.

### Sample size

The sample size for this trial was determined using *GPower *[58]. This indicated that complete data from 62 participants would provide 80% power to detect a minimal clinically important improvement of 20% in pain outcomes [49] for the full strength device using a one way analysis of variance (p = 0.05). This assumed a mean score of 65 on a standard 100 mm pain visual analogue scale (VAS) for the demagnetised device, with a difference of -13 for the full strength device and -6.5 for both the attenuated and copper devices, when compared with the demagnetised device. An upper limit of 21.7 mm was set for the common standard deviation within subjects. Estimates for both mean and standard deviation were based on data obtained by the MACROPOD trial, together with data presented by Segal and colleagues [32].

It is anticipated that loss to follow-up should not exceed 10%. In relation to this figure it is worthwhile noting that just 1 participant out of 45 (2%) withdrew from the MACROPOD trial. However the duration for participation (16 weeks) was shorter than the proposed trial. More importantly, participants in the CAMBRA trial will be asked to provide blood samples on successive occasions. This may serve as a disincentive for continued participation. Efforts will be made however to continue to follow up for participants who refuse to provide further blood samples. Allowing for a maximum of 10% attrition from recruitment to final data collection, 69 participants will need to be recruited.

### Recruitment

Initially the trial will seek to recruit six large general practices from three primary care trusts: 1) Hull Teaching PCT; 2) East Riding of Yorkshire PCT; 3) York & North Yorkshire PCT. The selection of larger practices (e.g. those having four or more partners) will help to ensure the availability of a practice nurse or phlebotomist at each site for the purpose of taking blood samples. Practice managers/practice staff will identify potentially eligible patients from medical records with support from the principal investigator. Administrative staff from each practice will then contact patients by post. Each patient will be sent an information sheet, letter of invite to participate and a standard covering letter expressing support for the trial, signed by one of the GPs. Patients will be asked to contact the principal investigator if they are interested in participation. Should the six initial practices fail to identify a sufficiently large number of patients with RA, then additional practices will be recruited.

**Figure 2:**
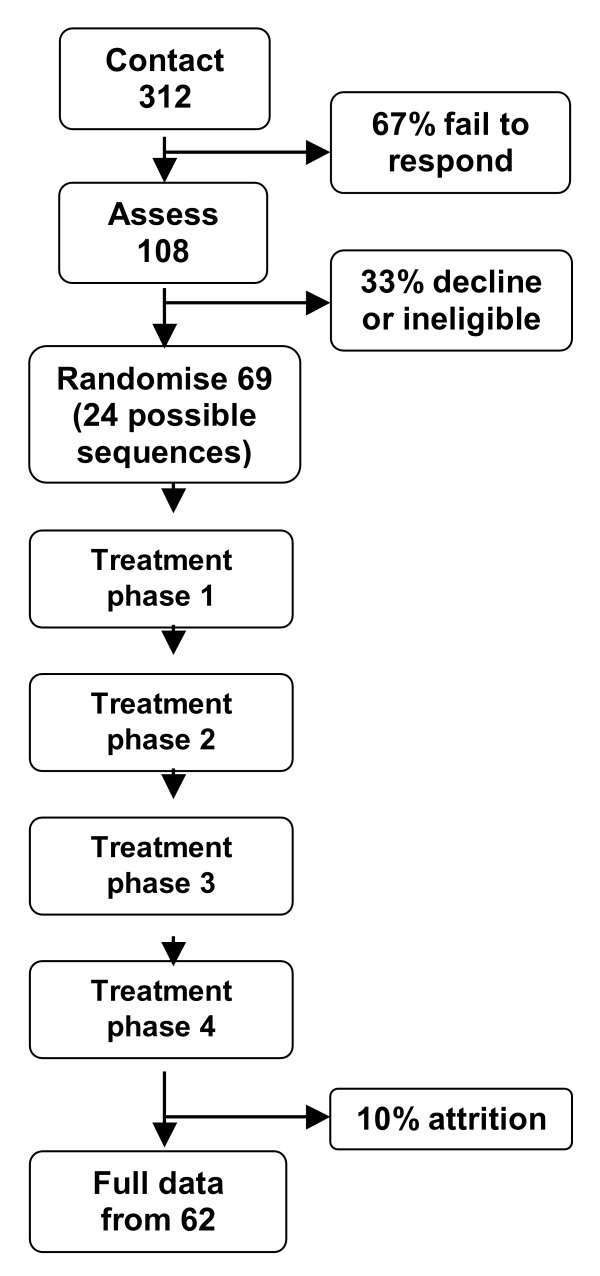
**Participant flow**. Illustration of anticipated CONSORT diagram, as used for the purpose of planning recruitment and data collection.

It is anticipated that approximately 312 potentially eligible patients with RA will need to be approached with information about the trial and invited to take part. Based on experience from previous trials and taking into consideration the belief that blood samples may serve as a disincentive for participation, it is expected that roughly two thirds (208) of patients will fail to respond (see Figure 2).

Patients who express an interest in taking part will receive an appointment for the principal investigator to visit them at home, or elsewhere if required. Appointment letters will be accompanied by a copy of the consent form and a baseline questionnaire. During the meeting the principal investigator will offer more information about the trial, show examples of the type of devices involved, and answer any questions. He will then assess eligibility, seek the patients' written consent to participate in the trial, and collect the baseline questionnaire. At this stage it is estimated that a further third (35) of patients will either be found to be ineligible or decline to participate. This should leave 69 patients who will then be entered into the trial. Meetings will normally take place within the patient's home as a pill count will be conducted if the person is recruited. General practices will be informed after each patient is recruited into the trial, and provided with four dates for the collection of blood samples.

### Planned analyses

Data from all participants recruited into the trial will be included the analyses according to intention to treat.

The main statistical method to be used will follow the basic estimators approach allowing for period effects as described by Senn [[43] pp.171–5]. This will involve testing for statistically significant differences in treatment outcomes amongst the four devices, based upon follow up scores from each treatment phase. Results will be reported for the following outcomes: pain; inflammation (CRP and PV values); swollen and tended joint counts; physical function; general health; medication use; coping and affect. Treatment effect estimates will be presented for each of the four devices together with their standard error and 95% confidence intervals. The computer package used for this purpose will be SPSS^® ^and possibly also SAS^®^.

If magnet therapy is of any true therapeutic benefit then one would expect to observe more favourable outcomes following use of experimental (full strength MagnaMax^®^) device, when compared with the other three control devices. Conversely, should a significant difference be observed with poorer outcomes for the demagnetised dummy device than the other three devices, then this would tend to indicate that any benefit of magnet therapy may be attributed simply to a placebo effect. This does however assume that most participants will identify the demagnetised as a placebo, but fail to recognise either the attenuated or copper bracelet as such.

Within the planned analyses a statistically significant difference (p < .05) in pain as measured by the VAS (as the primary outcome measure) will be viewed as the critical test of treatment effect. Whilst it is anticipated that sufficient data will be gathered in order to provide 80% power to detect a 20% relative improvement in pain VAS scores for the experimental device, it is nevertheless important to acknowledge that the pain VAS represents a rather simple instrument involving sizeable measurement error, as demonstrated by wide confidence intervals. Greater statistical power should therefore be provided by other more robust measures, such as the McGill Pain Questionnaire, which may offer greater sensitivity in terms of demonstrating differences in outcome between devices. Moreover I will seek to combine data gathered using a range of distinct clinical measures in order to form a single composite index of disease activity, as is recommended by the ACR, which will feature prominently within the planned analyses.

Whilst it may be possible to pretest data for evidence of carry over effects and to adjust effect estimates accordingly, this approach is probably unnecessary. Reasonable steps have been taken to account for possible carry over effects. In particular, the study design incorporates one week periods between treatment phase during which no device is worn, known as passive wash out periods. Moreover, the study design also incorporates further active wash out periods. Specifically, data is collected at the end of each five week treatment phase but not at the beginning. Together this would mean that any residual therapeutic effects following the removal of a device would need to persist for a total period of six weeks or more in order to affect the results of this study. This seems unlikely. Attempts to pretest data for carry over and to subsequently adjust for this may also be called into question. This approach assumes that no carry over effect has occurred if a statistical test fails to identify it. However the limited power of statistical tests used may simply offer false reassurance to the reader that there was no carry over effect, when the reverse may be true (type II error) [43].

Neither period effects nor period by treatment interactions are anticipated. Period effects refer a change between periods and within subjects which would have occurred independently from any treatment given. Plausible period effects in rheumatoid arthritis might arise from variations in symptoms corresponding to seasonal changes, a reduction in symptoms following diagnosis during a flare up (regression to the mean), or the widespread introduction of a new wonder drug. However the possible influence of such factors on treatment effect estimates obtained within the CAMBRA trial is most likely obviated by the use of a balanced randomisation procedure involving 24 unique treatment sequences, and the fact that participants will not all be recruited at the same time. Participants will also be recruited from databases held by general practices, not all of whom will be newly diagnosed or experiencing a flare up at the start of the trial. Nevertheless, it is difficult to anticipate all possible factors which might result in a period effect or period by treatment interaction. Therefore whilst the primary analysis will look at patient and treatment, I intend to test for period and period by treatment interactions using analysis of variance.

It is estimated that missing data will account for no more than 10% of questionnaires. However the proportion of missing data may be greater for blood test results, as this requires participants to visit their general practice. Missing blood test data will therefore be imputed where possible from questionnaire data available for each participant concerned. Unlike multiple imputation, this approach assumes that data might not be missing at random, which would be true if participants failed to provide blood samples only during periods when their symptoms flared up. Where no data whatsoever are available, then this will be left blank. Thereafter, sensitivity analysis will be performed in order to determine whether any missing data are related to treatment.

As a secondary step in the analysis, estimated treatment effects for each of the devices will be adjusted according to medication use, for which continuous data will be available. The assumption here is that this variable may serve to modify and otherwise mask the effects of treatment in terms of all major outcomes. For example, participants who benefit from magnet therapy may reduce their use of analgesic medication in response, and therefore demonstrate no apparent difference in terms of pain at follow up. The basic approach used for this purpose will be that of multiple regression, testing first for non-linear relationships [59].

Information gathered on compliance will also be used in order to perform an additional per protocol analysis, excluding relevant outcome data for those participants who failed to wear each device for the minimum recommended period. In pragmatic terms the results of this analysis are not especially important, however the may still be relevant to future research and of interest to readers. Differences in treatment effect may also be explored according to baseline variables including; level of education, belief in the therapeutic efficacy of magnetic and copper bracelets, and previous use of complementary and alternative medicine. Dangers associated with multiple testing will be acknowledged however. Information concerning success of blinding will be presented in detail and should aid interpretation of the findings.

Further refinement to analysis plan will be sought towards completion of the data collection stage of this trial. This will take into consideration advice from Professor Martin Bland. Statistical analyses will be performed at the end of the trial only, although adverse events and reactions will be actively monitored.

### Analyses of economic data

Quality of life, medication use and data relating to health care expenditure will be collected as part of the trial. If magnet therapy is shown to be effective for reducing either arthritis symptoms or medication use then a cost-effectiveness analysis will be conducted. This will be lead by staff from the Centre for Health Economics at The University of York.

### Treatment compliance

Non-compliance in terms of failure to wear devices for the recommended minimum of 12 hours per day is unlikely to represent a major problem. Compliance will be measured and systematic bias should be avoided due to the random allocation of participants to different intervention sequences. Whilst the present study employs placebo-control, the emphasis is nevertheless that of a pragmatic evaluation of the therapeutic effectiveness of magnet therapy within a real world setting [46]. Non-compliance therefore represents a valid outcome in its own right.

### Trial management

The Department of Health Sciences at The University of York will act as the co-ordinating centre for CAMBRA. The trial will be led by Stewart Richmond (SR) as the principal investigator, who will be responsible for clinical and scientific co-ordination, together with all aspects of day to day management. Professor David Torgerson (DT) will be responsible for managing the randomisation process, and will serve as PhD supervisor to SR, advising on matters of trial design and methodology. Professor Martin Bland (MB) will serve as a statistical advisor to the trial. Dr Hugh MacPherson (HM) will serve as an additional PhD supervisor to SR, and will advise on matters concerning the use and evaluation of complementary and alternative medicine. Professor Andrew Wong (UCLA, California) will also serve as an advisor on matters concerning rheumatology. It is anticipated that DT, MB and HM will also contribute towards the analysis and interpretation of data, and preparation of central publications arising from the trial.

A trial steering committee will be set up prior to recruitment in order to review proposals for the study and oversee progress. This will include academics, patient representatives and at least one general practitioner with an interest in the trial.

### Ethics and research governance

The trial will be conducted in accordance with revised Declaration of Helsinki and EC guidelines for Good Clinical Practice.

## Discussion

RA can be described as a common inflammatory musculoskeletal disease, which represents a significant burden to the UK health system and the economy. For the individual its effects are both distressing and disabling. Conventional drug treatments for RA may fail to control pain adequately and can present a risk to the individual. As a consequence, patients with arthritis sometimes seek complementary and alternative therapeutic approaches to pain management, such as magnet therapy. Historical underinvestment in CAM research has however resulted in a lack of scientific evidence concerning the therapeutic effectiveness of such devices. Further pragmatic research, such as that of the present trial, may therefore lead to innovative therapeutic advances in the cost-effective management of diseases such as RA. Within this context, the CAMBRA trial will seek to offer a meaningful scientific evaluation of the true therapeutic value of magnet therapy for RA using a novel methodological approach to overcome some of the inherent challenges encountered by research in this field.

Research on this topic appears particularly worthwhile given that conclusive findings either way would prove beneficial. If effectiveness can be demonstrated then magnet therapy may become a valuable addition to clinical practice, improving quality of life amongst patients and lowering demand for both consultations and specialist referrals. Moreover, it may serve to reduce reliance on potentially harmful drug treatments, thereby preventing unnecessary adverse events. In economic terms it is also worthwhile noting that one-off costs of providing magnetic devices are likely to be far lower than those of repeatedly prescribing analgesic medication. Therefore if, for example, drug prescription costs for arthritis and related conditions could be reduced by just 1% then this would result in an annual saving to the NHS of roughly £3.4 million [1]. Conversely, if this trial demonstrates that magnet therapy is ineffective then this may serve to educate patients and discourage further private expenditure on such device. Perhaps most importantly however the present study addresses an immediate need for guidance which will benefit health professionals and assist in making appropriate health care policy decisions.

### Trial update [August 2008]

At the time of submitting this protocol for publication the trial appears to be running well, and data collection will shortly be coming to an end. However delays have been encountered.

#### Study devices

Initial attempts were made to acquire suitable magnetic devices from Ecoflow plc [Cornwall, UK] between April and June 2006. This company is one of the leading distributors of magnetic bracelets in the UK. Recent findings from a well designed randomised placebo controlled trial appeared to demonstrate that Bioflow^® ^wrist straps, as supplied by this company, are effective for relieving pain amongst patients with osteoarthritis [33]. However errors in the manufacture of attenuated study devices, together with difficulties in blinding participants could have accounted for these findings [33,60]. It is all the more unfortunate therefore that Ecoflow plc refused to supply devices for further evaluation as part of the present trial.

In July 2006 MagnaMax Healthcare [Ontario, Canada] agreed to supply the trial with 210 MagnaMax^® ^magnetic wrists straps. These devices have been used widely within the UK and were studied previously within the MACROPOD trial. MagnaMax^® ^wrist straps are similar to those used in the study described above in terms of application and magnetic strength, and are fairly typical of the type of magnetic devices often worn by people with arthritis. Manufacturing costs for these devices were met by the trial. The devices were forwarded to Professor Kevin O'Grady and Beth Jones at the Physics Department of York University. Individual magnetic inserts were then given unique ID numbers and randomly allocated to one of three groups. The magnetic strength of each insert allocated to the first group (full strength) was then measured using a calibrated hall probe and recorded. These were found to have a maximum surface magnetic field strength of between 1502 and 2365 Oersteds. Inserts randomly allocated to the second (attenuated) and third (demagnetised) groups were individually subjected to reverse magnetic fields which reduced incrementally until all devices in the second group had a maximum surface field strength of between 250 to 350 Oersteds, and those in the third group had a residual magnetic field of less than 20 Oersteds.

**Figure 3:**
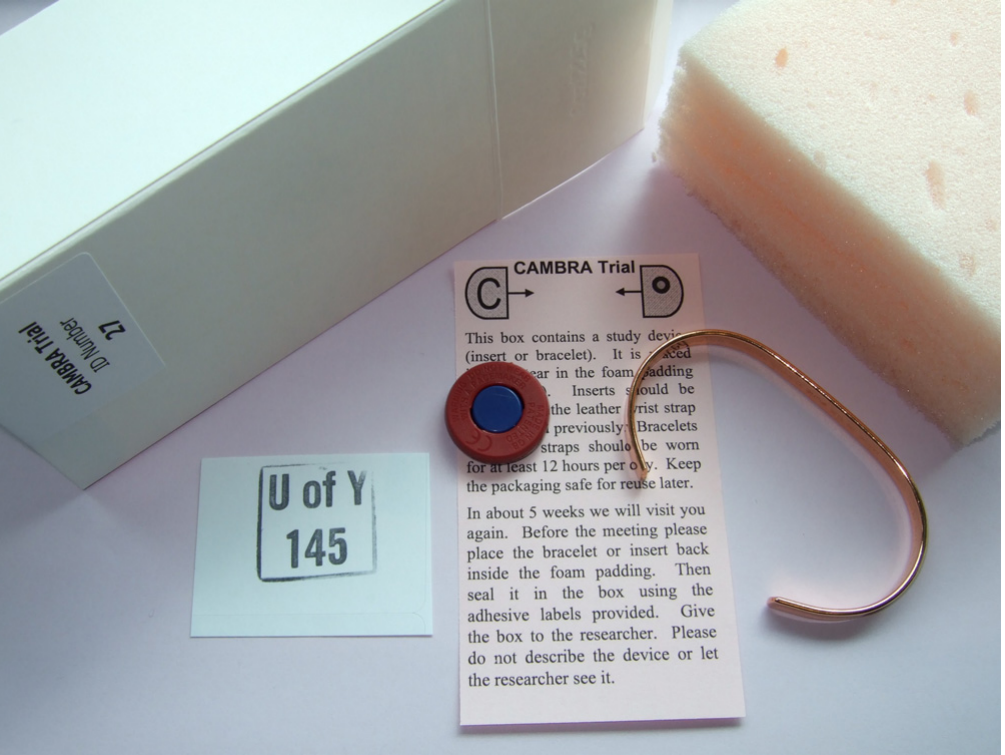
**Study devices and packaging**. Photograph of MagnaMax^® ^insert, copper bracelet, and materials used in packaging devices.

70 plain pure copper bracelets were purchased from a local pharmacy at trade cost. These together with the magnetic inserts were then passed on to Professor David Torgerson and Hannah Pearson at the York Trials Unit in order to be boxed and randomised according to the trial protocol [see Figure 3].

#### Regulatory approval

Full ethical approval for the trial was granted by Hull and East Riding Local Research Ethics Committee in February 2007, together with research governance approval shortly thereafter from Hull Teaching PCT, East Riding of Yorkshire PCT, and York & North Yorkshire PCT. Honorary contracts for the principal investigator were obtained from each of the NHS trusts concerned. Further R&D approval was secured for acute trusts as required.

#### Trial Steering Committee

A trial steering committee was formed in May 2007, which included academics from the University of York, two patient representatives from York Rheumatoid Arthritis Support Group and one external general practitioner. Proposals for the study were reviewed and passed with only minor alterations requested. The group agreed to meet in future to monitor progress.

#### Recruitment

Patient recruitment began in July 2007 and finished in March 2008. In total 70 people with RA were recruited, most of whom have now completed all aspects of the study. Participants were recruited from eight out of nine medical practices that had agreed to support the study.

Whilst sufficient numbers of patients were recruited into the trial in order to exceed the required sample size, the entire process of recruitment took longer than anticipated. This may be attributed to several factors, including an apparent reluctance amongst most medical practices approached to offer active support for the trial. Practice managers and general practitioners from 34 different medical practices were contacted in turn between June 2007 and January 2008 with written and verbal requests for support. Staff from 25 of these practices chose not to support the trial. Lack of available staff time was the most frequently cited reason for making this decision. Notably the timing of practice recruitment did coincide with a regional outbreak of norovirus together with the annual influenza vaccination programme. This appeared to have placed considerable strain the resources of general practices, and in particular the workload of practice nurses. The perceived absence of any meaningful financial incentive also appeared to have played a deciding role for a small number of practices that declined to help.

One further reason for extending the recruitment period was that there were fewer suitable patients within each practice than initially predicted, which led to the inclusion of additional practices from further afield. Fortunately, this was facilitated by the merger of several primary care trusts which happened just before the trial began. This extended the area for which the trial had regulatory approval. An application was also made for the trial to be included within the National Institute for Health Research (NIHR) clinical research network portfolio. This enabled the UK Primary Care Research Network to provided practical assistance by helping to recruit the final few practices.

#### Attrition and data collection

Of the 70 people recruited into the study one participant has died due to an unrelated medical condition and three others have withdrawn from the trial entirely. From the remainder, 56 people have finished all aspects of the trial and 10 others are currently completing the final treatment phases of the study. Excluding the four people mentioned initially, all participants have been successfully followed up after each treatment phase, providing complete questionnaire data to date. The exceptionally high completion rate for questionnaires observed so far is almost certainly attributable to the use of home visits as the main follow up method, which has helped to maintain commitment amongst participants and fostered trust with the principal investigator. Participants have also showed great enthusiasm for the study, which has been demonstrated by their overall willingness to have repeated blood samples taken at their general practice.

The inclusion of distant practices has made running the trial somewhat more complex than expected, requiring collaboration from additional haematology and biochemistry departments across different acute trusts. Data collection has also proved to be highly time consuming due to the need for repeated home visits in a large geographical area across which some participants live more than 100 miles apart.

## Abbreviations

ACR: American College of Rheumatology; CAM: Complementary and alternative medicine; CAMBRA: Copper And Magnetic Bracelets for Rheumatoid Arthritis: a randomised placebo-controlled crossover trial; CRP: C-reactive protein; DMARD: Disease modifying anti-rheumatic drugs; ESR: Erythrocyte sedimentation rate; MACROPOD: Magnetic And Copper bracelets for the Relief Of Pain in Osteoarthritis: a randomised Double blind placebo-controlled trial; NHS: National Health Service; NSAID: Non steroidal anti-inflammatory drug; PV: Plasma viscosity; PCT: Primary care trust; RA: Rheumatoid arthritis; RCT: Randomised controlled trial; RESPECT: Randomised Evaluation of Shared Prescribing for Elderly people in the Community over Time; VAS: Visual analogue scale.

## Competing interests

The author declares that they have no competing interests.

## Authors' contributions

SR designed the study for the purpose of his ongoing PhD research. He obtained funding and study devices, secured ethics and research governance approval, and wrote the study protocol. He is responsible preparing all trial materials, recruitment of practices and patients, follow-up of patients enrolled into the study, analysis and interpretation of data, and dissemination of findings.
